# Toward a Hybrid Intrusion Detection Framework for IIoT Using a Large Language Model

**DOI:** 10.3390/s26041231

**Published:** 2026-02-13

**Authors:** Musaad Algarni, Mohamed Y. Dahab, Abdulaziz A. Alsulami, Badraddin Alturki, Raed Alsini

**Affiliations:** 1 Department of Computer Science, Faculty of Computing and Information Technology, King Abdulaziz University, Jeddah 21589, Saudi Arabia; malqarni0476@stu.kau.edu.sa (M.A.); mdahab@kau.edu.sa (M.Y.D.); 2 Department of Information Systems, Faculty of Computing and Information Technology, King Abdulaziz University, Jeddah 21589, Saudi Arabia; aaalsulami10@kau.edu.sa; 3 Department of Information Technology, Faculty of Computing and Information Technology, King Abdulaziz University, Jeddah 21589, Saudi Arabia; baalturki@kau.edu.sa

**Keywords:** Industrial Internet of Things (IIoT), intrusion detection system (IDS), Large Language Model (LLM), classification (CLS), Principal Component Analysis (PCA)

## Abstract

The widespread connectivity of the Industrial Internet of Things (IIoT) improves the efficiency and functionality of connected devices. However, it also raises serious concerns about cybersecurity threats. Implementing an effective intrusion detection system (IDS) for IIoT is challenging due to heterogeneous data, high feature dimensionality, class imbalance, and the risk of data leakage during evaluation. This paper presents a leakage-safe hybrid intrusion detection framework that combines text-based and numerical network flow features in an IIoT environment. Each network flow is converted into a short text description and encoded using a frozen Large Language Model (LLM) called the Bidirectional Encoder Representations from Transformers (BERT) model to obtain fixed semantic embeddings, while numerical traffic features are standardized in parallel. To improve class separation, class prototypes are computed in Principal Component Analysis (PCA) space, and cosine similarity scores for these prototypes are added to the feature set. Class imbalance is handled only in the training data using the Synthetic Minority Over-sampling Technique (SMOTE). A Random Forest (RF) is used to select the top features, followed by a Histogram-based Gradient Boosting (HGB) classifier for final prediction. The proposed framework is evaluated on the Edge-IIoTset and ToN_IoT datasets and achieves promising results. Empirically, the framework attains 98.19% accuracy on Edge-IIoTset and 99.15% accuracy on ToN_IoT, indicating robust, leakage-safe performance.

## 1. Introduction

The Industrial Internet of Things (IIoT) is the foundation of modern cyber–physical systems (CPSs) that drive the developments of Industry 4.0 in critical infrastructure, production and intelligent environments [1,2]. The total IIoT sector is projected to reach $1.1 trillion by 2028 [3], and tens of billions of connected devices are projected by 2030 [4]. This integration of continuous sensing, networking and management allows for higher performance effectiveness. However, this extensive connectivity exposes these vital networks to an evolving cyberattack environment [3,4]. The risks in IIoT security are particularly high, as breaches can result in not only compromised data integrity and unauthorized access, but also physical device compromises, production system interruption and major security challenges [5]. For example, more than 12,000 attacks on smart home systems were detected in a single week [6], which highlights the critical need for adequate security measures. Recent industry analysis indicates a growing integration of traditional SCADA architectures and IoT-enabled frameworks, which increases the relevance of intrusion detection mechanisms for cyber–physical and IoT systems. This integration provides enhanced performance, but simultaneously introduces increased cybersecurity risks, necessitating the development of next-generation detection methods [7]. Furthermore, recent analysis of industrial datasets reveals that Denial-of-Service (DoS) and Distributed Denial-of-Service (DDoS) attacks are among the most prevalent threats, with DoS traffic accounting for 89.98% of recorded attack traffic in IIoT environments. This volume illustrates the high number of compromised devices being leveraged to disrupt the global network and potentially turn off production systems [5]. As a result, the design and implementation of robust intrusion detection systems (IDSs) are considered critical security techniques, especially in resource-constrained IIoT settings where real-time analysis and reduced computational cost are necessary [6,8].

Recent studies highlight significant challenges in the use of Machine Learning (ML) and deep learning (DL) for IIoT/IoT IDS. Datasets generated by these networks often include heterogeneous data types (e.g., categorical traffic fields and unstructured raw payload data) and have a high dimensionality of features, which requires manual feature engineering and robust model training [6,8]. Additionally, real-world IIoT datasets often show a significant class imbalance, with considerably more benign traffic than malicious attack instances, which leads to incorrect models that choose the majority class and fail to detect unusual but critical attack patterns [7,9]. Furthermore, inadequate validation methods in previous work, such as not considering temporal limitations or performing preprocessing like scaling or oversampling on the entire dataset before splitting, can cause data leakage, which results in incorrect evaluation of model performance and compromised security in practical implementation [10].

Moreover, data leakage occurs when information from the test set unintentionally influences the training process, leading to overly optimistic results that do not generalize in practice. In IIoT IDSs, leakage commonly arises when preprocessing steps—such as scaling, feature selection, or resampling—are applied to the entire dataset before the train and test split. In addition, it occurs when duplicate flows from the same traffic source or capture context appear across splits. This is particularly problematic under class imbalance, because oversampling can propagate test-set patterns into the training space and inflate performance metrics [10].

To address these ongoing challenges, research has changed rapidly to advanced hybrid and deep learning frameworks. Specifically, Bidirectional Encoder Representations from Transformers (BERT) is categorized as a Large Language Model (LLM). Such models, which are deep neural networks typically based on the transformer architecture, have emerged as a promising method for encoding sequence and semantic patterns in raw network traffic, moving beyond concentrating on individually designed numerical features [4,6,8]. The development of efficient and lightweight hybrid models is now essential, which combines deep temporal feature extraction with resource-aware classification pipelines [6]. Previous work has demonstrated the efficacy of coupling dimensionality reduction techniques such as Principal Component Analysis (PCA) with oversampling strategies such as the Synthetic Minority Oversampling Technique (SMOTE) to manage high dimensionality and class imbalance before training ensemble models [7,8]. However, an integrated system that effectively integrates the contextual feature power of an LLM with the structural efficiency of feature engineering and the robustness of tree-based ensembles, which ensures leakage safety across both heterogeneous modalities and complex threat landscapes, remains an active research area. In addition, our framework enforces split-specific processing: we first split the data, then fit all transformations on the training split only (scaling, PCA, prototype construction, and Random Forest (RF)-based Top-*K* selection), and apply the learned transforms to the held-out test split without re-fitting. Likewise, SMOTE is applied exclusively on the training split. This protocol yields a leakage-safe evaluation that better reflects real deployment behavior.

In this work, we propose a hybrid network traffic intrusion detection framework designed particularly for a secure and effective IIoT environment. Our approach combines text-derived and numerical features and leverages a frozen encoder-only BERT architecture to generate semantic embeddings from network flow features. The main contributions of this paper are summarized as follows.

- We enhance the feature representation by computing cosine similarity scores between each sample and class prototypes in PCA space. These similarity scores are added to the feature vector and used as input to tree-based classifiers.
- We address class imbalance only in the training split using SMOTE, which ensures that there is no projected bias.
- We use an RF for efficient selection of top features, followed by a robust Histogram-based Gradient Boosting (HGB) classifier to produce final predictions.
- We evaluate the proposed framework on two publicly available IIoT datasets, namely Edge-IIoTset and ToN_IoT. The experiments show that the resulting detection pipeline shows high performance while maintaining leakage-safe validation strategies, which are important for reliable evaluation on the considered IIoT datasets.

The rest of this paper is structured as follows: Section 2 discusses related work, the proposed methodology is explained in Section 3, experimental results are reported in Section 4, and conclusions are drawn in Section 5.

## 2. Related Works

In this section, we review recent related works in IDSs for the Internet of Things and Industrial Internet of Things networks, focusing on the models, techniques and datasets employed across several studies. The field is characterized by efforts to leverage the ML and DL paradigms and is often validated using heterogeneous and challenging datasets such as ToN_IoT and Edge-IIoTset. Table 1 is a summary of related work, which shows the authors, the year of publication, the methods or techniques used, the datasets used and the accuracy achieved.

Dhirar and Hamad [11] conducted a comprehensive evaluation of a generated intrusion detection system (IDS) dataset tailored for Software-Defined Networking (SDN)–IoT environments named SDN-IoT. Their study compared this dataset with three used datasets, namely BoT-IoT, ToN_IoT and InSDN, using four DL architectures, including Convolutional Neural Networks (CNNs), Long Short-Term Memory (LSTM), Recurrent Neural Networks (RNNs) and deep neural networks (DNNs). The SDN-IoT dataset was generated using Mininet-WiFi, Ryu, HOIC, LOIC, Hping3 and CICFlowMeter. They achieved 98.48% accuracy using DNNs on the SDN-IoT dataset, 86.94% accuracy using LSTM on InSDN and 75.00% accuracy using LSTM on ToN_IoT. Maseno et al. [12] proposed a hybrid feature reduction technique that integrates CNNs, LSTM and attention to improve feature selection for IoT intrusion detection. After feature reduction, they applied Support Vector Machine (SVM) and RF classifiers with SMOTETomek used for data balancing. Using the ToN_IoT dataset, their approach achieved 98% accuracy with Random Forest and 91% accuracy with SVM. Salehiyan et al. [3] introduced an optimized hybrid DL framework known as transformer–GAN-AE that is designed for intrusion detection in edge and industrial IoT environments. The model combines transformer, GAN and autoencoder components and leverages the Improved Chimp Optimization Algorithm (IChOA) for hyperparameter tuning. They evaluated on the WUSTL-IIoT-2021, Edge-IIoTset and ToN_IoT datasets; the framework achieved an accuracy rate of 97.86%, 98.63% and 98.92% respectively. Ngo et al. [13] proposed a Top-K Similarity Graph Framework (TKSGF) for IoT intrusion detection that constructs graphs based on attribute level similarity rather than physical network structure. The use of cosine similarity to build the graphs and the use of GraphSAGE as the Graph Neural Network (GNN) model compared to Graph Convolutional Networks (GCNs) and Graph Attention Networks (GAT)) demonstrated good performance. On the NF-ToN_IoT dataset, GraphSAGE achieved an F1-score of 100% for binary classification and on NF-BoT_IoT achieved an F1-score of 98.52%.

**Table 1:** Summary of existing works.

Authors & Ref	Year	Methods/Techniques	Dataset(s)	Best Accuracy
Dhirar and Hamad [11]	2025	Deep Learning (CNN, LSTM, RNN, DNN). Dataset Generation.	SDN-IoT (Custom), BoT-IoT, ToN_IoT, InSDN.	SDN-IoT (Custom): 98.48%
Maseno et al. [12]	2024	Hybrid Feature Reduction (CNN-LSTM–Attention), Classification (SVM, RF).	ToN_IoT Datasets.	RF: 98%
Salehiyan et al. [3]	2025	Transformer–GAN-AE with Improved Chimp Optimization Algorithm (IChOA).	WUSTL-IIoT-2021, Edge-IIoTset, ToN_IoT.	97.86%, 98.63%, 98.92%.
Cao et al. [14]	2025	FedDynST (FL + APPNP Graph CNN + 1D-CNN).	CICDDoS2019, Edge-IIoTset.	Edge-IIoTset: 97.28%
Ismail et al. [15]	2025	ML/Ensemble (DT, RF, LGBM, Stacking). FS (MI).	ToN_IoT, WUSTL-IIOT-2021, Edge-IIoTset.	DT: 96.25%
Sadhwani et al. [16]	2025	CNN, LSTM, BiLSTM with SHAP-based XAI feature selection.	NSL-KDD, UNSW-NB15, ToN_IoT, X-IIoTID.	98.21%, 92.9%, 97.80%, 98.09%
Alqura’n et al. [17]	2024	ANNs (BLNN, TLNN). FS (MI, RFE).	NF-ToN_IoT-v2, Edge-IIoTset.	NF-TON_IoT-v2: 99.84%
Qathrady et al. [18]	2024	Self-Attention + CNN (SACNN). FS (ETC).	Edge-IIoTset, X-IIOTID.	99.95%, 99.81%
Anwer et al. [19]	2025	Hybrid DL (CNN-Bi-LSTM) + Federated Learning (FL).	X-IIOTID, WUSTL-IIoT, Edge-IIoTset.	97.8%, 95.4%, 96.2%
Alshehri et al. [20]	2024	Self-Attention (SA) + DCNN. Preprocessing (MI/Cleaning).	IoTID20, Edge-IIoTset.	96.89%, 99.95%
Abdulkareem et al. [21]	2024	FS (FI/ETC), Stack Ensemble Learner (SEL) (DT, NB, LR).	Edge-IIoTset.	87.37%

Cao et al. [14] developed a dynamic spatiotemporal deep learning solution called FedDynST to detect DDoS attacks in collaborative cloud–edge ICS environments. The system integrates federated learning with dynamic weights, APPNP-based graph convolutional networks and 1D-CNN techniques to construct feature graphs and analyze traffic data. FedDynST achieved an accuracy of 97.28% on the CICDDoS2019 dataset and accuracy of 96.28% on Edge-IIoTset. Ismail et al. [15] performed a comparative analysis of multiple lightweight supervised ML algorithms to determine whether the models are suitable for resource-constrained IoT and IIoT systems. Using Decision Trees (DTs)), RF, and ensemble learning methods such as bagging, stacking, and LightGBM—with Mutual Information for feature selection—the study tested models on TON-IoT, WUSTL-IIoT-2021, and Edge-IIoTset. The results indicated good performance, with LightGBM achieving 97.8% Micro-F1 on TON-IoT and Decision Trees achieving 96.25% accuracy on WUSTL-IIoT-2021 using transfer learning. Alqura’n et al. [17] introduced a new approach to detect XSS attacks in IoT systems operating on 5G networks using Artificial Neural Networks (ANNs)). The method evaluates narrow, bilayered and trilayered ANN architectures and applies filter- and wrapper-based feature selection methods such as Mutual Information and RFE. Tested on NF-TON-IoT-v2 and Edge-IIoTset, the approach achieved high accuracy, including 99.84% accuracy with a bilayered ANN and 99.79% accuracy using a trilayered ANN. Qathrady et al. [18] developed SACNN-IDS, a self-attention-based Convolutional Neural Network designed to detect intrusions in IIoT networks. The framework incorporates a self-attention mechanism with a CNN architecture and uses an extra tree classifier for feature extraction. It was evaluated on the Edge-IIoTset and X-IIOTID datasets, and SACNN-IDS achieved a high accuracy rate of 99.95% on Edge-IIoTset and an accuracy rate of 99.81% on X-IIOTID.

Anwer et al. [19] proposed a hybrid CNN–Bi-LSTM DL framework integrated with federated learning to improve intrusion detection performance in IIoT settings. The model uses SMOTE for data balancing and was evaluated on X-IIOTID, WUSTL-IIoT and Edge-IIoTset. In centralized settings, the model achieved up to 97.8% accuracy on X-IIOTID and 96.2% accuracy on Edge-IIoTset, while the federated deployment achieved slightly lower but still good results. Alshehri et al. [20] presented a Self-Attention-based Deep CNN (SA-DCNN) for intrusion detection in IIoT networks. Their approach incorporates a self-attention layer with a DCNN and uses a two-step preprocessing strategy to remove intraclass and cross-class duplicates, followed by Mutual Information-based feature filtering. SA-DCNN achieved an accuracy rate of 96.89% on IoTID20 and an accuracy rate of up to 99.96% on Edge-IIoTset. Abdulkareem et al. [21] proposed a lightweight ensemble learning framework called FI-SEL for the detection of IoT and IIoT attacks. The method combines feature importance-based dimensionality reduction using the extra tree classifier with a stacked ensemble learner, which comprises DT, Naïve Bayes (NB) and logistic regression (LR) classifiers. Using only eight features from the Edge-IIoTset dataset, FI-SEL achieved an accuracy of 87.37%. Sadhwani et al. [16] integrated eXplainable Artificial Intelligence (XAI) into a deep learning IDS for IoT by training CNN, LSTM, and BiLSTM models on NSL-KDD, UNSW-NB15, ToN_IoT, and X-IIoTID and then using SHAP to select the 15 most influential features per dataset. Retraining with these reduced feature sets preserved or slightly improved performance, achieving 98.21% (NSL-KDD), 97.80% (ToN_IoT), 92.90% (UNSW-NB15), and 98.09% (X-IIoTID). Although this improves training efficiency and interpretability, SHAP-based feature subsets remain dataset-specific, and their cross-dataset transferability or suitability for constrained edge deployment has not been evaluated.

Despite these advances, many existing studies do not fully address both textual and numerical feature fusion within a single framework while also applying leakage-safe validation through split-specific preprocessing. In addition, many recent IDS approaches rely on heavily fine-tuned or complex deep models, which can increase computational cost and raise the risk of overfitting, especially when IIoT datasets are imbalanced or prone to data leakage.

## 3. Materials and Methods

### 3.1. Proposed Architecture

**Figure 1:**
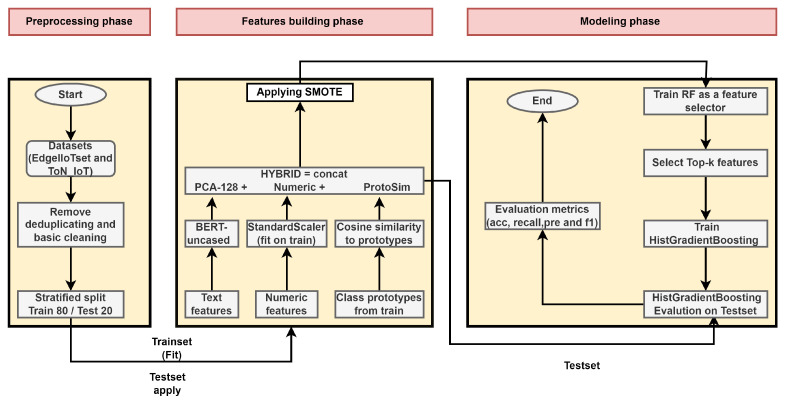
Overall proposed architecture including the three phases: preprocessing, feature building and modeling.

Our framework introduces hybrid network flow intrusion detection by combining textual and numerical features, as shown in Figure 1. The architecture comprises three phases: preprocessing (Section 3.2.1), feature building (Section 3.2.2), and modeling (Section 3.2.5). The procedure begins with public IoT datasets (ToN_IoT and Edge-IIoTset). Using public IoT datasets affects the framework in two ways. First, the pipeline is general by design because it relies on common flow fields (protocol, IPs, and ports) plus basic numeric traffic features, with optional metadata when available. Therefore, it can be applied to other flow-based IoT/IIoT data with small changes, mainly by mapping column names and choosing the available text fields in the template. Second, we use ToN_IoT and Edge-IIoTset to make the experiments reproducible and easy to compare with prior work. The same feature construction and leakage-safe training steps can also be used in practice when only flow records and limited metadata are available. In real deployments, results may change with traffic conditions, encryption, and label quality, so the framework can be retrained or calibrated using site-specific data when needed.

In feature building, flow records are rendered as short text strings and paired with numeric traffic statistics. An LLM-based encoder provides a fixed text embedding, which is compressed by PCA to d = 128; numeric fields are standardized; and train-only class prototypes are used to produce prototype similarity scores. We fix the PCA bottleneck to 128-d and the RF budget to 128-d a priori to balance fidelity and efficiency. However, compressing the 768-d to 128-d provides a six-times reduction without degrading accuracy. In addition, limiting selection to K = 128 prevents any single branch from dominating the hybrid vector. These components are concatenated into a compact, fully numeric hybrid vector. To address the imbalanced classes in each dataset, we utilize SMOTE to balance the datasets.

In modeling, an RF is used to select the top informative features and train a lightweight tree-based classifier on the selected hybrid representation. For the evaluation, HGB was utilized to classify the selected hybrid features, leveraging efficient, high-accuracy boosting on tabular data. All trainable transforms and models are fit on the training split only; the held-out test split is never used for fitting—its flows pass through the frozen BERT and the train-fitted transforms to produce test features.

### 3.2. Dataset Description

The selection of appropriate datasets is critical for the rigorous evaluation of experimental designs and implementations. After surveying a broad body of prior work and empirical results, we adopted two widely used datasets for the network flow IDS: ToN_IoT and Edge-IIoTset. These datasets were selected to cover complementary IoT operating contexts and to enable a fair comparison against a large body of IDS literature. ToN_IoT provides heterogeneous telemetry from realistic IoT/IIoT services and includes diverse attack types with notable class imbalance, making it suitable for evaluating performance under imbalanced classes. Edge-IIoTset, in contrast, focuses on edge-centric IoT environments and offers rich flow-level features across multiple protocols and devices, allowing us to evaluate robustness under edge traffic dynamics. Importantly, both datasets are publicly available, widely used in recent IDS studies, and support reproducible network-flow evaluation.

ToN_IoT [22] provides 44 engineered network flow features and covers IoT-centric threats such as ransomware, backdoor attacks, scanning, Distributed Denial of Service (DDoS), Denial of Service (DoS), data injection, Cross-Site Scripting (XSS), and Man-In-The-Middle (MITM); it also includes zero-day traces collected from heterogeneous IoT devices and distributed test-lab (DTL) nodes.

Edge-IIoTset [23] targets IoT and IIoT environments and offers 61 network flow features spanning threats such as DDoS on Hypertext Transfer Protocol (HTTP), Transmission Control Protocol (TCP), User Datagram Protocol (UDP), and ICMP Internet Control Message Protocol (ICMP), Structured Query Language (SQL) injection, MITM, ransomware, port scanning, vulnerability scanning, password attacks, uploading, fingerprinting, and XSS.

#### 3.2.1. Preprocessing Phase

The preprocessing stage normalizes text fields, standardizes numeric fields, and fixes the train and test protocol before any fitting. The same steps are applied to both datasets.

1. Dataset loading: Load one CSV per dataset into a dataframe and drop exact duplicate rows.
2. Text field representation: Select the available protocol and application text fields, normalize them (trim/lowercase and remove placeholders), and then concatenate them with protocol, IP, and port tags into a single domain-aware string per flow (collapse whitespace).
3. Numeric casting and Booleanization: For a fixed list of numeric candidates, coerce values to numeric. Map Boolean-like fields deterministically to (0, 1).
4. Stratified split (80/20): Shuffle and partition each dataset into non-overlapping train/test subsets (80/20) using class-stratified sampling so that per-class proportions are preserved in both splits.

#### 3.2.2. Feature Building Phase

**Figure 2:**
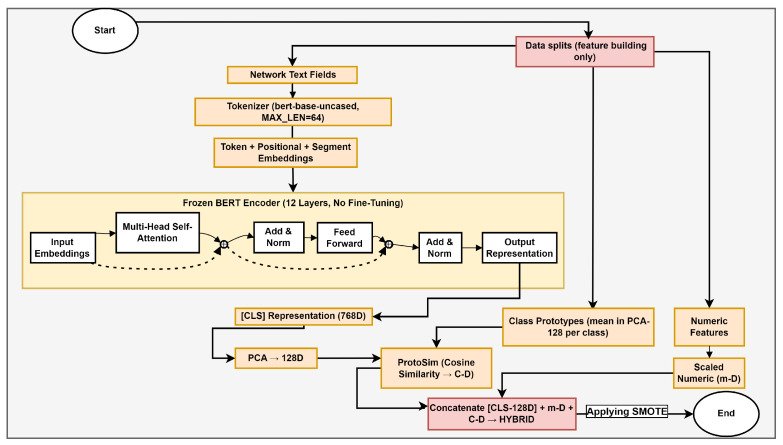
Feature building phase: flow—fusing text-derived embeddings.

Figure 2 details how heterogeneous flow records are transformed into a compact feature vector. We render protocol, service, DNS, and HTTP fields as short text via a template and pair them with tabular traffic statistics. The text branch utilizes a 12-layer bidirectional transformer, serving as a deterministic feature extractor without fine-tuning; the tabular branch standardizes numeric fields. All steps with equations are clarified below.

#### 3.2.3. Large Language Model-Based Encoder

BERT is an LLM-based encoder pre-trained with masked-language modeling and next-sentence prediction, enabling each token to attend bidirectionally to both left and right context and thereby learn rich, general-purpose representations [24].

In addition, BERT has recently been utilized to classify attacks in IDSs due to its ability to detect complex attack patterns in datasets Ferrag et al. [4]. Several recent studies leverage BERT’s self-attention-based contextual encoding to extract semantic representations of network flows, achieving strong intrusion detection performance [25,26,27]. BERT first converts text into token and subword embeddings via tokenization. Each sequence is framed with the special tokens Classification (CLS) and Separator (SEP) to make it suitable for the encoder. In our pipeline, we use a frozen BERT (bert-base-uncased) purely as a deterministic feature extractor for network-flow-to-text strings. This choice is motivated by the nature of IoT text (URIs, DNS names, user-agents, and MQTT topics), where WordPiece tokenization confers robustness to rare or fragmented tokens, and by the need to avoid overfitting and reduce compute in edge-oriented IDSs. In this phase, we use various techniques to build and reduce the dimensionality of data. Concretely, after encoding we retain only the final hidden state of the (CLS) token (a 768-dimensional vector) as the text representation; all token-level embeddings are discarded and (SEP) serves only as a boundary marker.The (CLS) vector is then passed to downstream steps (e.g., dimensionality reduction and fusion) as the sole text feature. The following equations follow the standard transformer definitions commonly used in recent IoT IDS studies [28].

In tokenization and input embeddings, each network flow record is first converted into a short sentence and then tokenized with a pre-trained AutoTokenizer (WordPiece, MAX_LEN = 64) to produce token, learned positional, and segment embeddings. As shown in Equation (1), for a tokenized flow $w_{i,1:T_{i}}$, the encoder input at step *t* is

(1) $$e_{i,t}=E\left(w_{i,t}\right)+P\left(t\right)+S\left(seg_{t}\right),$$

where *E*, *P*, and *S* are token, positional, and segment embeddings, respectively.

Table 2 provides the exact flow-to-text construction used in our implementation, including (i) the concatenation order (template) and (ii) the complete list of text fields for each dataset. It also clarifies that the representation is payload-free and discusses the impact of TLS.

In the process, we use protocol/service/state fields, protocol/IP/port tags, and only application metadata strings when present in the dataset (e.g., HTTP method/URI, DNS query name, and MQTT topic/message). Consequently, purely metadata-based representations may be less sensitive to subtle content-driven application layer attacks. Under TLS encryption, some HTTP strings can be unavailable; in such cases, the text representation falls back to transport/network tags and available TLS handshake metadata, while the numeric features still capture statistical traffic behavior.

After the tokenization and input embedding step, the token sequence ${\left\{e_{i,t}\right\}}_{t=1}^{T_{i}}$ is fed to a BERT encoder whose parameters are kept fixed (no fine-tuning). Self-attention is computed using the learned projections $Q=XW^{Q}$, $K=XW^{K}$, and $V=XW^{V}$, with the scaled dot-product attention

(2) $$\mathrm{Attn}\left(Q,K,V\right)=\mathrm{softmax}\;\left(\frac{QK^{⊤}}{\sqrt{d_{k}}}\right)V.$$

With *H* heads, the encoder applies multi-head attention followed by residual connections and layer normalization:

(3) $$\begin{array}{cc} U & =\mathrm{LayerNorm}\;\left(X+\mathrm{MultiHead}(X)\right), \end{array}$$

(4) $$\begin{array}{cc} Y & =\mathrm{LayerNorm}\;\left(U+\mathrm{FFN}(U)\right), \end{array}$$

where FFN is a position-wise feed-forward network (Dense → GELU → Dense). We initialize the encoder input as $X^{\left(0\right)}=E+P+S$ and denote the encoder output by $H=X^{\left(L\right)}$. The downstream text embedding is the sequence-level summary at (CLS):

(5) $$h_{i}^{\mathrm{CLS}}\;=\;H_{i,(CLS)}\in R^{768}.$$

#### 3.2.4. Dimensionality Reduction

We apply PCA to the high-dimensional embeddings before forming the hybrid feature vector. PCA retains the directions with the largest variance, which suppresses noise and weak signals, mitigates the curse of dimensionality, and reduces the risk of overfitting. It also decorrelates features and improves numerical conditioning, making downstream steps—SMOTE, RF-based feature selection, and HGB training—more stable and efficient in both time and memory.

In practice, to quantify potential information loss when compressing frozen BERT (CLS) embeddings from 768 to 128 dimensions, we fit PCA on the training split only and evaluate retention using complementary tests. PCA-128 achieves a Cumulative Explained Variance (CEV) of 99.31%, indicating that most embedding variance is preserved. We further assess reconstruction fidelity via inverse transformation, obtaining a reconstruction MSE of $6.46\times 10^{-5}$. In addition, cosine retention between original embeddings and their reconstructions is 0.999878±0.00062 (mean ± std), confirming near-perfect angular preservation. Table 3 reports PCA-128 retention and efficiency metrics computed with train-only PCA fitting for two datasets.

Let $\mu_{pca}$ and $U_{128}\;\in \;R^{768\times 128}$ denote the mean and top-128 principal directions. Each CLS is compressed as shown in Equation (6).

(6) $$z_{i}\;=\;U_{128}^{⊤}\;\left(h_{i}^{CLS}-\mu_{pca}\right)\;\in \;R^{128}.$$

To address numeric features, assume $x_{i}^{\mathrm{num}}={\left(x_{i,1},\ldots ,x_{i,m}\right)}^{⊤}$, with train means $\mu_{j}$ and stds $\sigma_{j}>0$, as shown in Equation (7).

(7) $${\tilde{x}}_{i,j}\;=\;\frac{x_{i,j}-\mu_{j}}{\sigma_{j}}\;,\;j=1,\ldots ,m,$$

forming the standardized vector ${\tilde{x}}_{i}^{\mathrm{num}}={\left({\tilde{x}}_{i,1},\ldots ,{\tilde{x}}_{i,m}\right)}^{⊤}$.

In the PCA-128 space, compute per-class prototypes from the dataset. Equation (8) identifies if $S_{c}$ is the train set of class c∈{1,…,C}:

(8) $$p_{c}\;=\;\frac{1}{|S_{c}|}\sum_{i\in S_{c}}z_{i}\;\in \;R^{128}.$$

For any sample *i*, the ProtoSim (cosine similarity) scores for all prototypes are shown in Equation (9).

(9) $$s_{i,c}\;=\;\frac{z_{i}^{⊤}p_{c}}{∥z_{i}{∥}_{2}\;{∥p_{c}∥}_{2}}\;,\;s_{i}={\left(s_{i,1},\ldots ,s_{i,C}\right)}^{⊤}\in R^{C}.$$

Finally, we concatenate the PCA-CLS, standardized numeric, and ProtoSim components into the hybrid feature consumed by tree-based classifiers, which is defined in Equation (10).

(10) $$\phi_{i}\;=\;\left[\;z_{i}\;\;;\;\;{\tilde{x}}_{i}^{\mathrm{num}}\;\;;\;\;s_{i}\;\right]\in R^{128+m+C}.$$

We employ SMOTE rebalancing to mitigate the class imbalance present in both datasets on the training split only, leaving the test split unchanged for evaluation. Given the training hybrid matrix $\Phi_{\mathrm{train}}\in R^{n_{\mathrm{tr}}\times \left(128+m+C\right)}$ and labels $y_{\mathrm{train}}\in {\left\{1,\ldots ,C\right\}}^{n_{\mathrm{tr}}}$, let $n_{c}=\left|S_{c}\right|$ and $n_{\max}=\max_{c}n_{c}$. We set the classwise target size as in Equation (11); for each minority class with $n_{c}<n_{c}^{\mathrm{tgt}}$, we synthesize $\Delta_{c}=n_{c}^{\mathrm{tgt}}-n_{c}$ samples via *k*-NN interpolation as in Equation (12). The resulting training set after SMOTE is summarized in Equation (13).

(11) $$n_{c}^{\mathrm{tgt}}\;=\;\max\;\left\{\;n_{c},\;\left\lceil \rho \;n_{\max}\right\rceil \right\}.$$

(12) $$\tilde{\phi }\;=\;\phi_{i}\;+\;\lambda \;\left(\phi_{j}-\phi_{i}\right),\;i\in S_{c},\;j\in N_{k}\left(i\right)\cap S_{c},\;\lambda ∼U\left(0,1\right),$$

where $N_{k}\left(i\right)$ is the set of *k* nearest neighbors of *i* (Euclidean) within class *c*.

(13) $$\left(\Phi_{\mathrm{train}}^{\mathrm{sm}},\;y_{\mathrm{train}}^{\mathrm{sm}}\right)\;=\;\mathrm{SMOTE}\;\left(\Phi_{\mathrm{train}},\;y_{\mathrm{train}};\;\rho ,\;k\right).$$

We use PCA (6), the scaler (7), and prototypes (8) on the training split only. During testing, these transforms and the tokenizer of BERT are kept fixed and used to obtain $\phi_{i}$ via (10), with no re-estimation on test data.

#### 3.2.5. Modeling Phase

**Figure 3:**
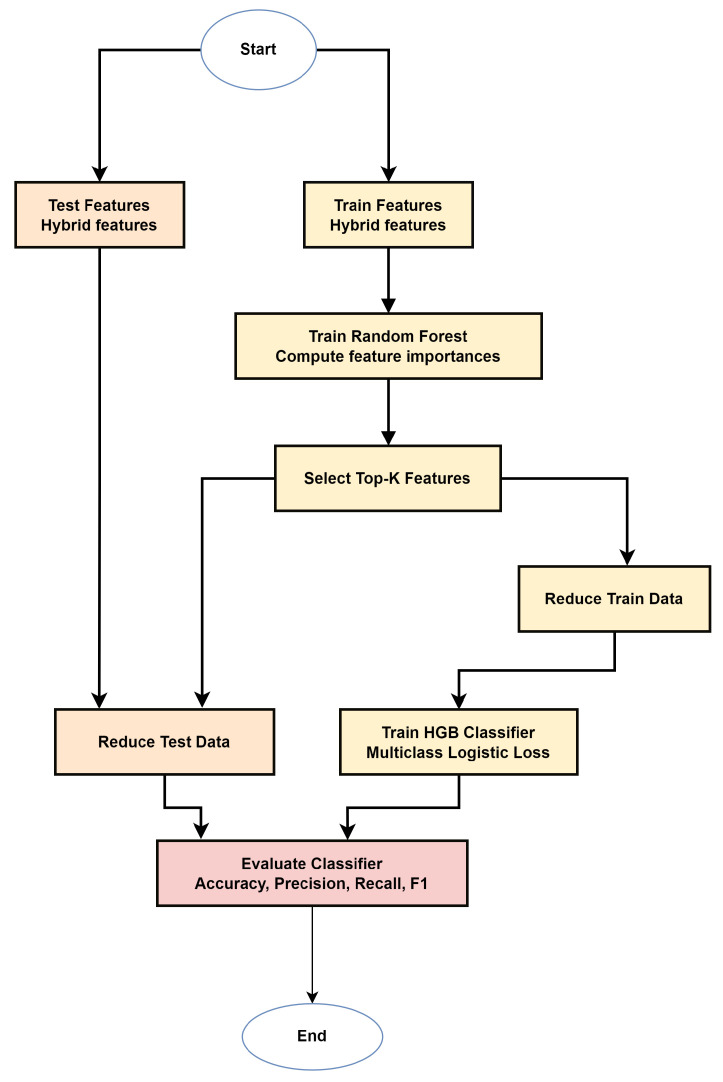
Modeling phase diagram.

This phase consumes the hybrid features from Section 3.2.2 to build a compact multiclass classifier. As shown in Figure 3, we (i) perform RF-based top-128 feature selection on the training split and (ii) train an HGB model; we then (iii) evaluate on the held-out test split.

Let $\Phi_{\mathrm{train}}\;\in \;R^{n_{\mathrm{tr}}\times d}$ and $\Phi_{\mathrm{test}}\;\in \;R^{n_{\mathrm{te}}\times d}$ denote the hybrid matrices with labels $y_{\mathrm{train}}\;\in \;{\left\{1,\ldots ,C\right\}}^{n_{\mathrm{tr}}}$. A Random Forest fitted on $\left(\Phi_{\mathrm{train}},y_{\mathrm{train}}\right)$ yields impurity-based importances $\gamma \;\in \;R^{d}$. RF selects the Top-*K* features via Equation (14),

(14) J=TOPK(γ,K),

and forms the reduced matrices in Equation (15),

(15) $$\Phi_{\mathrm{train}}^{\mathrm{sel}}=\Phi_{\mathrm{train}}\left[:,J\right],\;\Phi_{\mathrm{test}}^{\mathrm{sel}}=\Phi_{\mathrm{test}}\left[:,J\right].$$

We then train HGB on $\left(\Phi_{\mathrm{train}}^{\mathrm{sel}},y_{\mathrm{train}}\right)$ using the multiclass logistic objective, as summarized in Equation (16).

(16) $$L_{\mathrm{CE}}\;=\;-\frac{1}{n_{\mathrm{tr}}}\sum_{i=1}^{n_{\mathrm{tr}}}\log p_{F}\;\left(y_{\mathrm{train},i}|\Phi_{\mathrm{train},i}^{\mathrm{sel}}\right),$$

where $p_{F}\left(\cdot |\cdot \right)$ are the class probabilities produced by the trained HGB model F. Eventually, we evaluate the model on the test set and compute the performance of metrics.

We follow the hybrid pipeline in Algorithm 1 and the three phases; all operators and settings are exactly as defined there.
**Algorithm 1** Hybrid feature pipeline for network flow IDS**Input:** Data F, labels *y*, tokenizer *T*, frozen BERT *M*, numeric set N, PCA dim d=128, SMOTE (ρ,k), Top–*K*, split *r*.**Output:** Test metrics (accuracy, precision, recall, $F_{1}$) and predictions $\hat{y}$.  1: **Load & split**: read F, drop duplicates; stratified split *r* into train/test.  2: **Flow→Text**: build short sentence per flow via protocol/IP/port tags.  3: **Frozen BERT**: encode text with *M*; take fixed [CLS] embedding (768-d).  4: **PCA (train only)**: fit on train CLS; project all to d=128.  5: **Numeric (train only)**: select N, cast booleans, fit StandardScaler on train; transform all.  6: **Prototypes (train only)**: compute class centroids in PCA space; get ProtoSim (cosine) scores to all prototypes.  7: **Hybrid feature**: concatenate [PCA-CLS; ProtoSim; standardized numeric].  8: **SMOTE (train only)**: rebalance hybrid-train with (ρ,k).  9: **RF Top–*K***: train RF on hybrid-train (SMOTE); select best *K* features.10: **Train HGB**: fit HistGradientBoosting on selected hybrid-train.11: **Evaluate**: predict on selected hybrid-test; report accuracy, precision, recall, $F_{1}$, and confusion matrix.

### 3.3. Experimental Setup

Table 4 summarizes the configuration used in all experiments. A frozen bert-base-uncased provides (CLS) features; PCA (128-D) compresses text embeddings; standardized numeric features and prototype similarity scores are concatenated; RF selects the top-128; and an HGB classifier is trained. All transforms are fit on the training split only; the test split is used once for final reporting.

### 3.4. Evaluation Metrics

We assess detection performance using four standard metrics—accuracy, precision, recall, and F1-score well suited to cybersecurity [29], where both false alarms and missed attacks carry operational risk. Let TP, TN, FP, and FN denote the confusion matrix counts.

(17) $$\mathrm{Accuracy}=\frac{TP+TN}{TP+TN+FP+FN}$$

(18) $$\mathrm{Precision}=\frac{TP}{TP+FP}$$

(19) $$\mathrm{Recall}=\frac{TP}{TP+FN}$$

(20) $$F1\text{-}\mathrm{score}=\frac{2\;\mathrm{Precision}\cdot \mathrm{Recall}}{\mathrm{Precision}+\mathrm{Recall}}$$

## 4. Results and Discussion

We report the proposed work in terms of accuracy, precision, recall, and F1-score, along with the confusion matrix and per-class ROC/PR curves.

### 4.1. Performance Metrics

**Figure 4:**
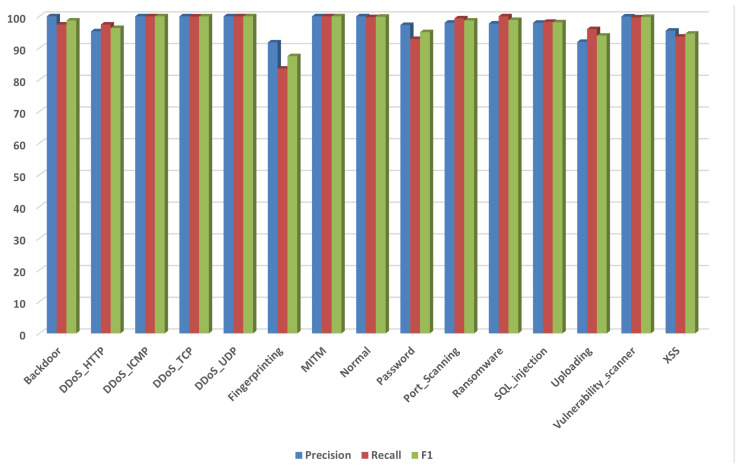
Edge-IIoTset: Recall, precision, and F1-score.

For the Edge-IIoTset, as depicted in Figure 4, the grouped bars indicate a consistently strong detector, with an overall accuracy of 98.10%, macro-F1 of 97.40%, and weighted-F1 of 98.10%. Several high-impact classes achieve very high performance, such as DDoS_ICMP and DDoS_UDP with F1-score = 100.00%, DDoS_TCP with F1-score = 99.98%, and Vulnerability_scanner with F1-score = 99.80%, while normal traffic is near perfect with F1-score = 99.87%. The comparatively lower bars are concentrated in fingerprinting with F1-score = 87.43% and recall = 83.50%, uploading with F1-score = 93.90%, and XSS with F1-score = 94.53%, consistent with residual confusions among semantically adjacent HTTP-like behaviors. Generally, the results indicate robust decision boundaries with minimal error accumulation across classes.

**Figure 5:**
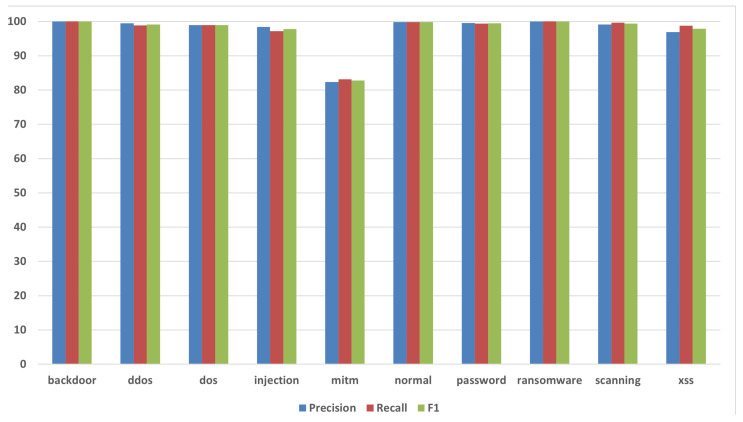
ToN_IoT: Recall, precision, and F1-score.

On ToN_IoT, as illustrated in Figure 5, performance is high and uniform on the held-out test set: accuracy of 99.10%, macro-F_1_ of 97.50%, and weighted-F_1_ of 99.10%. Several categories are essentially saturated, including backdoor with F1-score = 99.99% and ransomware with F1-score = 99.98%; normal, password, and scanning also remain near the top with F1-score = 99.84%, 99.47%, and 99.41%, respectively. The remaining classes maintain strong scores—for example, DDoS with F1-score = 99.17%, DoS with F1-score = 98.97%, injection with F1-score = 97.77%, and XSS with F1-score = 97.86%. The single clearly weaker class is mitm with F1-score ≈ 82.78%, which is expected given its small support. Overall, the near-ceiling bars across most classes indicate robust separability and stable generalization.

### 4.2. Confusion Matrices

**Figure 6:**
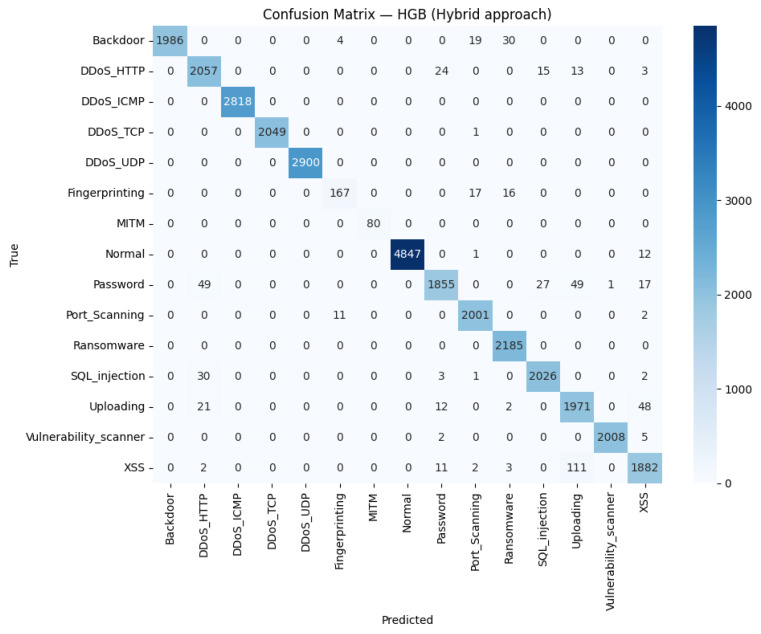
Edge-IIoTset confusion matrix for the hybrid model.

For Edge-IIoTset in Figure 6, the confusion matrix shows that correct predictions clearly outweigh mistakes across almost all classes, with the darkest cells where the predicted label matches the true class. Misclassifications are few and mostly confined to behaviorally related categories—such as occasional mix-ups among password, Port_Scanning, and uploading—together with a small spillover from fingerprinting into normal. This pattern suggests that false alarms and missed detections are rare and localized rather than widespread. In practice, the model draws strong boundaries for the major DoS families and routine traffic, while the remaining errors likely reflect overlapping traffic cues or limited support in minority subsets. Overall, the figure supports that the hybrid representation provides discriminative signals with minimal cross-class contamination.

**Figure 7:**
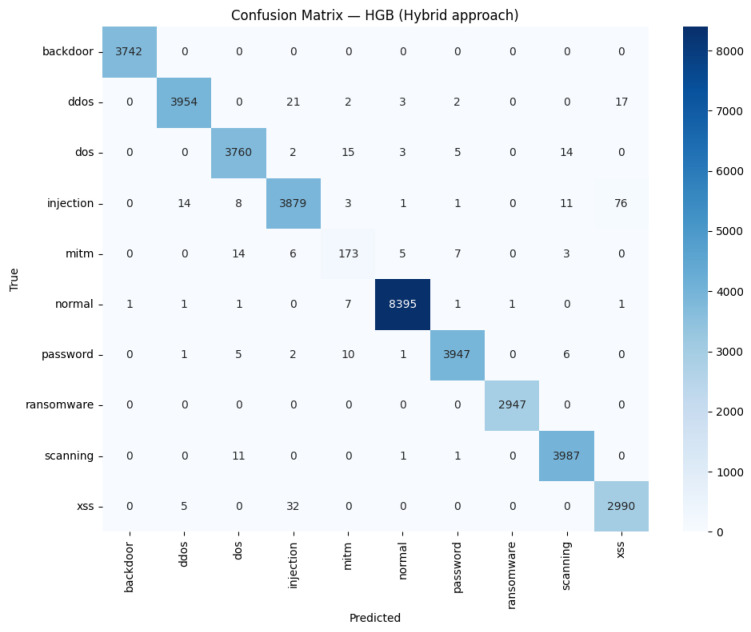
ToN_IoT confusion matrix for the hybrid model.

For ToN_IoT in Figure 7, a similar picture emerges: most classes are classified correctly far more often than not, and the visible errors are small in number and tightly clustered. The main confusions arise in minority categories—most notably limited bleed from MITM and injection into neighboring labels—whereas frequent classes such as normal, password, and scanning remain highly stable. This concentration of mistakes is consistent with class imbalance effects rather than a systematic issue in the representation. Operationally, the matrix indicates reliable behavior on a heterogeneous benchmark, and it suggests that further gains would come from targeted data enrichment or prototype sharpening for the rare, semantically adjacent classes.

### 4.3. ROC Curves

**Figure 8:**
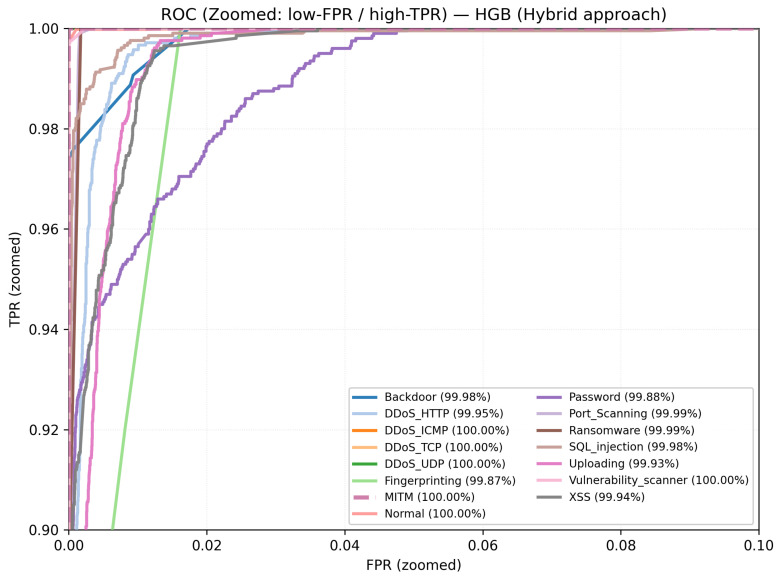
Edge-IIoTset: One-vs-rest ROC curves for the hybrid model.

For Edge-IIoTset in Figure 8, the one-vs-rest ROC curves cluster near the top-left corner, indicating very high true positive rates at low false positive rates across nearly all attack classes. The separation is consistent for both volumetric DoS variants and routine traffic. Overall, the curves remain concentrated near the upper-left region of the ROC space, reflecting strong separation between positives and negatives across classes. They also indicate near-unity per-class AUCs, supporting a robust model that remains reliable under heterogeneous traffic and class imbalance.

**Figure 9:**
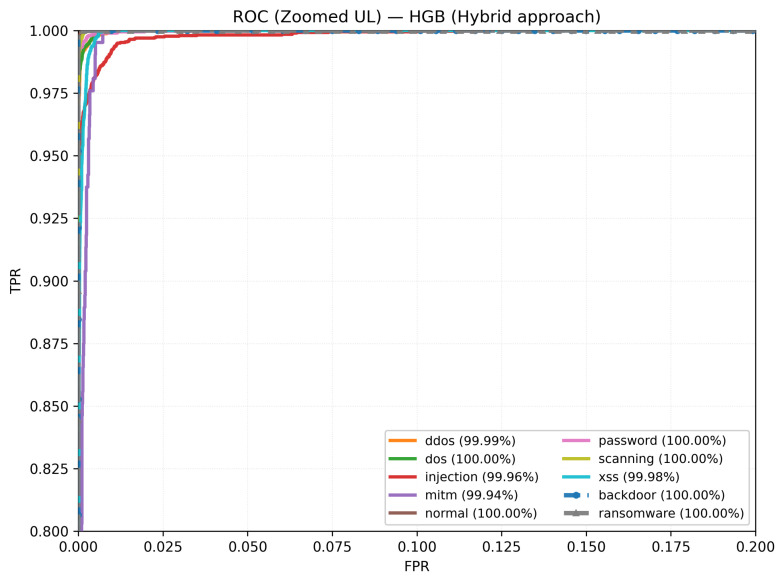
ToN_IoT: One-vs-rest ROC curves for the hybrid model.

For ToN_IoT in (Figure 9), the curves likewise rise sharply, with most classes achieving near-perfect discrimination. Residual deviations appear mainly in minority categories, but the operating region stays close to high TPR with low FPR, supporting reliable detection under heterogeneous traffic and label imbalance. Most traces remain tight and high along the ROC frontier even at low false positive rates, reflecting the reliable separability of the frequent categories. Class-wise AUCs are consistently high for the majority classes, with minor dips confined to rare classes—evidence of stable generalization under class imbalance. In particular, the slight dip for MITM is consistent with the confusion matrix, where MITM is a minority class (208 samples) and shows limited overlap with neighboring behaviors (173 correctly classified; most errors map to DoS (14), injection (6), normal (5), and password (7)). Such confusions are expected under a payload-free representation, where MITM deviations can be subtle compared with more distinctive volumetric attacks.

### 4.4. Ablation Study

To quantify the contribution of each feature branch, we ran an ablation on Edge-IIoTset and ToN_IoT under the same split protocol and classifier settings. The held-out test results are summarized in Table 5 (Edge-IIoTset) and Table 6 (ToN_IoT); accuracy is overall, and precision/recall/F1-score are support-weighted.

As reported in Table 5, numeric-only already achieves strong results (accuracy ≈94.61%), clearly surpassing text-only (accuracy ≈84.93%), which shows that handcrafted traffic statistics are highly informative. The hybrid approach is best across all metrics (accuracy ≈98.19%), adding ∼3.58 percentage points over numeric-only and ∼13.27 over text-only in accuracy. This indicates that the hybrid approach provides complementary evidence that tightens decision boundaries and reduces residual errors.

Table 6 shows that text-only lags (accuracy ≈90.11%), while numeric-only is near ceiling (accuracy ≈98.84%). The hybrid approach adds a consistent, albeit smaller, gain over numeric-only (about +0.32 percentage points in accuracy) and a large gain over text-only (about +9.04 percentage points). This pattern suggests that (i) numerical traffic statistics dominate on ToN_IoT and (ii) integrating them with PCA-CLS and ProtoSim still improves ranking and separation, especially for challenging or minority labels.

### 4.5. Comparison Study

As summarized in Table 7, prior work on Edge-IIoTset is dominated by deep learning approaches and stacked ensembles. Transformer–GAN–AE by Salehiyan et al. [3] combines a transformer, GAN, and autoencoder and reaches 98.63% accuracy and 98.79% recall on Edge-IIoTset. FedDynST by Cao et al. [14], which couples federated learning with APPNP-based graph convolution and a 1D–CNN, attains 97.28% accuracy, 97.14% precision, 91.28% recall, and 97.62% F1-score. Self-attention CNN family models yield the numerically strongest results: SACNN–IDS by Qathrady et al. [18] reports around 99.95% accuracy, while SA–DCNN by Alshehri et al. [20] slightly improves this to 99.96%. In contrast, the FI–SEL stack by Abdulkareem et al. [21], which combines decision trees, Naive Bayes, and logistic regression on only eight selected features, achieves 87.37% accuracy, 90.65% precision, 77.73% recall, and 80.88% F1-score on the same dataset.

Within this landscape, our hybrid approach on Edge-IIoTset attains 98.19% accuracy, 98.21% precision, 98.19% recall, and 98.19% F1-score. Although slightly below the best self-attention CNN variants in raw accuracy, it is competitive with the optimized transformer–GAN–AE model and improves on FedDynST in all four metrics (about +0.91 percentage points in accuracy, +1.07 in precision, +6.91 in recall, and +0.57 in F1-score). Compared to the FI–SEL ensemble, the hybrid HGB narrows the gap to the top-performing deep models while offering much stronger recall and F1-score (gains of roughly +20.46 and +17.31 percentage points, respectively). These results show that a tree-based classifier fed with our hybrid representation can match or exceed several sophisticated deep architectures on Edge-IIoTset, while maintaining balanced accuracy, precision, recall, and F1-score rather than optimizing a single metric.

For ToN_IoT, the literature again spans diverse designs. The hybrid feature reduction pipeline of Maseno et al. [12] achieves 98.00% accuracy, 93.00% precision, and 98.00% recall on ToN_IoT. Transformer–GAN–AE by Salehiyan et al. [3] reaches 98.92% accuracy and 99.52% recall on the same dataset. The lightweight LightGBM baseline of Ismail et al. [15] reports 97.80% accuracy, precision, recall, and F1-score. In addition, Sadhwani et al. [16] employs CNN, LSTM, and BiLSTM models with SHAP-based feature selection and reports 97.80% accuracy on ToN_IoT after reducing each dataset to 15 influential features, while shortening training time and improving interpretability. In contrast to the Edge-IIoTset setting, the ToN_IoT baselines in Table 7 exhibit complementary strengths, but no single method simultaneously dominates the others in terms of accuracy, precision, recall, and F1-score.

On ToN_IoT, our hybrid approach achieves 99.15% accuracy, 99.16% precision, 99.15% recall, and 99.15% F1-score, which are the highest values among the methods listed in Table 7, including the XAI-based deep models of Sadhwani et al. [16]. The hybrid HGB improves over the transformer–GAN–AE accuracy by about 0.23 percentage points and over the 97.80% baselines of Ismail et al. [15] and Sadhwani et al. [16] by about 1.35 percentage points, while simultaneously providing a complete set of accuracy, precision, recall, and F1-score. Relative to Maseno et al. [12], our model increases precision from 93.00% to 99.16% and raises the F1-score accordingly, indicating fewer misclassifications while retaining high coverage of positive instances.

Overall, the comparison highlights two main strengths of our work. First, the same hybrid approach architecture—combining PCA-CLS and ProtoSim features with standardized numerics—is evaluated consistently on both Edge-IIoTset and ToN_IoT, whereas many existing studies focus on a single dataset or report only a subset of metrics (reflected by the NR entries). Second, despite using a tree-based HGB classifier rather than a dedicated deep CNN or transformer encoder, our model attains performance that is (i) competitive with the best deep learning methods on Edge-IIoTset and (ii) superior to all reported baselines on ToN_IoT in terms of the joint accuracy, precision, recall, and F1-score. This suggests that the proposed hybrid representation is highly effective at capturing discriminative structure across heterogeneous IIoT traffic.

### 4.6. Inference Latency of the Decision Stage

To address feasibility on IoT devices, we report lightweight inference-time measurements for the final decision stage. Specifically, we measure the latency of HGB on the final test feature vector (after the frozen BERT embedding, train-only PCA, ProtoSim, and Top-*K* selection). Timings are computed on the test split and averaged over repeated runs to reduce noise. The PCA retention and projection-cost analysis is reported separately in Table 3.

For Edge-IIoTset, the measured HGB inference time is 0.031 ms/sample. For ToN_IoT, the HGB inference time is 0.026 ms/sample as illustrated in Table 8. Overall, these results indicate that the post-embedding classifier stage is computationally inexpensive and can support low-latency operation when embeddings are available.

Moreover, it is important to note that the above measurement targets the decision-stage computation only (post-embedding), which reflects the cost of the final classifier once the feature vector is formed. In practical settings, frozen embeddings may be pre-computed per flow, cached, or computed on nearby devices. However, end-to-end deployment latency will also depend on the embedding extraction hardware, batching strategy, and system. The small latency difference between Edge-IIoTset and ToN_IoT is expected and can be attributed to dataset-specific feature distributions. In addition, it can be attributed to sparsity patterns after top-128 selection, which influence the number of active histogram bins evaluated during inference. Overall, these results suggest that the post-embedding classification stage is computationally inexpensive and can help enable responsive IDS operation once embeddings are available. Nonetheless, full end-to-end deployment time remains system-dependent.

## 5. Conclusions

In conclusion, we presented an IDS framework for IIoT network flows that fuses complementary signals. First, frozen-transformer text embeddings are obtained by rendering flows as short phrases and encoding them once. Second, standardized numerical traffic statistics and prototype similarity scores were computed in PCA space from training data only. These components are concatenated into a single numeric vector and consumed by tree-based learners after SMOTE balancing and RF top selection. All transforms (tokenizer, PCA, scaler, and prototypes) are fitted strictly on the training split and kept fixed thereafter, ensuring leakage-safe evaluation and reproducibility. Across two heterogeneous benchmarks, the framework attains high and uniform performance of 98.19% on Edge-IIoTset and 99.15% on ToN_IoT by accuracy and weighted F1-score. Confusion matrices show that errors are sparse and localized, while one-vs-rest ROC curves concentrate near ideal regions for most classes. The ablation study confirms that the hybrid representation consistently outperforms text-only and numeric-only variants, indicating that text-derived context and prototype cues add non-redundant information to conventional traffic features without requiring any fine-tuning of the language model. Practically, the design is attractive for IIoT: the encoder is frozen BERT, dimensionality is controlled by PCA, and inference relies on lightweight tree models, making the approach easy to deploy and reproduce. A practical limitation of this work is the absence of a full deployment study on heterogeneous edge hardware. Although we report lightweight inference-time measurements for the decision stage under our experimental setup, end-to-end deployment behavior—including embedding extraction, batching, and system I/O—may vary across devices. Future work will therefore focus on detailed performance evaluation on representative edge gateways to more accurately assess real-time feasibility. In addition, it will explore light domain adaptation on top of the frozen encoder to better handle novel tokens. Furthermore, it will incorporate temporal or graph context to complement the current per-flow view while profiling latency on edge hardware.

## Figures and Tables

**Table 2:** Flow-to-text construction per dataset (payload-free): exact template and full field list.

Dataset	Flow→Text Template/Fields (No Raw Payload)
ToN_IoT	Template (concatenation order): [PROTO={proto}] [SERVICE={service}] [STATE={conn_state}] [SRC={src_ip}] [DST={dst_ip}] [SPORT={src_port}] [DPORT={dst_port}] + optional_app_metadata optional_app_metadata (when available): [DNS={dns_query}] [TLS_VER={ssl_version}] [CIPHER={ssl_cipher}] [SUBJ={ssl_subject}] [ISSUER={ssl_issuer}] [HTTP_M={http_method}] [URI={http_uri}] [HTTP_VER={http_version}] [UA={http_user_agent}] [HTTP_MIME_O={http_orig_mime_types}] [HTTP_MIME_R={http_resp_mime_types}] [WEIRD={weird_name}] [WEIRD_A={weird_addl}] Columns used: proto, service, conn_state, dns_query, ssl_version, ssl_cipher, ssl_subject, ssl_issuer, http_method, http_uri, http_version, http_user_agent, http_orig_mime_types, http_resp_mime_types, weird_name, weird_addl
Edge-IIoTset	Template (concatenation order): [PROTO={proto}] [SRC={ip.src_host}] [DST={ip.dst_host}] [SPORT={tcp.srcport/udp.port}] [DPORT={tcp.dstport/udp.port}] + optional_app_metadata optional_app_metadata (when available): [HTTP_M={http.request.method}] [FULL_URI={http.request.full_uri}] [QUERY={http.request.uri.query}] [REFERER={http.referer}] [DNS={dns.qry.name}] [MQTT_P={mqtt.protoname}] [TOPIC={mqtt.topic}] [MSG={mqtt.msg}] [MSG_DEC={mqtt.msg_decoded_as}] Columns used: http.request.method, http.request.full_uri, http.request.uri.query, http.referer, dns.qry.name, mqtt.protoname, mqtt.topic, mqtt.msg, mqtt.msg_decoded_as

**Table 3:** PCA-128 retention and efficiency metrics across datasets (PCA fit on the training split only).

Metric	Edge-IIoTset	ToN_IoT
PCA components	128	128
CEV (%)	99.32	99.66
Reconstruction MSE	$6.46\times 10^{-5}$	$2.87\times 10^{-5}$
Cosine retention (mean ± std)	0.999878±0.000620	0.999946±0.000361

**Table 4:** Configuration used in all experiments.

Parameter	Setting
Hardware	Intel Core i7-11800H, 32 GB RAM, Windows 64-bit; GPU available and used for BERT embedding extraction
Software	Python (3.10.11) with PyTorch (2.5.1+cu121) and Hugging Face Transformers (4.45.2); scikit-learn (1.2.2) for PCA/SMOTE/RF/HGB
Data split	80/20 stratified, seed 42
Text encoder	bert-base-uncased (frozen), MAX_LEN = 64, batch = 32
PCA (text)	128-D (fit on train)
Numeric scaling	StandardScaler (fit on train)
Prototype similarity	Cosine for per-class prototypes (train only)
SMOTE	target ratio ρ=0.85, neighbors k=3 (train only)
RF Top-K	K=128; trees = 600; bootstrap
Classifier (HGB)	Learning rate 0.06; iterations 500; max depth 12; $L_{2}=10^{-4}$; early stopping
Evaluation	Accuracy, precision, recall, $F_{1}$; confusion matrix and per-class ROC/PR

**Table 5:** Ablation study on Edge-IIoTset (held-out test split).

Model	Accuracy (%)	Precision (%)	Recall (%)	F1-Score (%)
Text-only	84.93	85.86	84.93	84.20
Numeric-only	94.61	94.80	94.61	94.63
Hybrid approach	98.19	98.21	98.19	98.19

**Table 6:** Ablation study on ToN_IoT (held-out test split).

Model	Accuracy (%)	Precision (%)	Recall (%)	F1-Score (%)
Text-only	90.11	91.71	90.11	89.55
Numeric-only	98.84	98.85	98.84	98.84
Hybrid approach	99.15	99.16	99.15	99.15

**Table 7:** Comparison of reported IDS performance on Edge-IIoTset and ToN_IoT, including our proposed HGB hybrid model. NR = not reported.

Study	Year	Dataset	Accuracy (%)	Precision (%)	Recall (%)	F1-Score (%)
Edge-IIoTset						
Salehiyan et al. [3]	2025	Edge-IIoTset	98.63	NR	98.79	NR
Cao et al. [14]	2025	Edge-IIoTset	97.28	97.14	91.28	97.62
Qathrady et al. [18]	2024	Edge-IIoTset	99.95	99.79	99.80	99.79
Alshehri et al. [20]	2024	Edge-IIoTset	99.96	99.83	99.79	99.81
Abdulkareem et al. [21]	2024	Edge-IIoTset	87.37	90.65	77.73	80.88
Hybrid approach	2025	Edge-IIoTset	98.19	98.21	98.19	98.19
ToN_IoT						
Maseno et al. [12]	2024	ToN_IoT	98.00	93.00	98.00	NR
Salehiyan et al. [3]	2025	ToN_IoT	98.92	NR	99.52	NR
Sadhwani et al. [16]	2025	ToN_IoT	97.80	NR	NR	NR
Ismail et al. [15]	2025	ToN_IoT	97.80	97.80	97.80	97.80
Hybrid approach	2025	ToN_IoT	99.15	99.16	99.15	99.15

**Table 8:** HGB inference latency on the final test features (post-embedding stage).

Metric	Edge-IIoTset	ToN_IoT
HGB inference (ms/sample)	0.031	0.026

## Data Availability

All datasets used in this study are publicly available and can be accessed in the cited sources (Edge-IIoTset and ToN_IoT).
